# Carbohydrate-dense snacks are a key feature of the nutrition transition among Ghanaian adults – findings from the RODAM study

**DOI:** 10.29219/fnr.v65.5435

**Published:** 2021-05-06

**Authors:** Frauke Assmus, Cecilia Galbete, Sven Knueppel, Matthias B. Schulze, Erik Beune, Karlijn Meeks, Mary Nicolaou, Stephen Amoah, Charles Agyemang, Kerstin Klipstein-Grobusch, Silver Bahendeka, Joachim Spranger, Frank P. Mockenhaupt, Liam Smeeth, Karien Stronks, Ina Danquah

**Affiliations:** 1 Department of Molecular Epidemiology, German Institute of Human Nutrition Potsdam-Rehbruecke, Nuthetal, Germany;; 2 Unit of Epidemiology, Statistics and Mathematical Modelling, Department of Exposition, German Federal Institute for Risk Assessment (BfR), Berlin, Germany;; 3 Department of Public Health, Amsterdam UMC, University of Amsterdam, Amsterdam Public Health Research Institute, Amsterdam, The Netherlands;; 4 Institute of Tropical Medicine and International Health, Charité - Universitaetsmedizin Berlin, Corporate Member of Freie Universitaet Berlin, Humboldt-Universitaet zu Berlin, and Berlin Institute of Health, Berlin, Germany;; 5 Julius Global Health, Julius Center for Health Sciences and Primary Care, University Medical Center Utrecht, Utrecht University, Utrecht, The Netherlands;; 6 Division of Epidemiology & Biostatistics, School of Public Health, Faculty of Health Sciences, University of the Witwatersrand, Johannesburg, South Africa;; 7 St. Francis Hospital Nsambya, Uganda Martyrs University, Kampala, Uganda;; 8 Department of Endocrinology and Metabolism, DZHK (German Center for Cardiovascular Research) partner site Berlin, Center for Cardiovascular Research (CCR), Charité – Universitaetsmedizin Berlin, Corporate Member of Freie Universitaet Berlin, Humboldt-Universitaet zu Berlin, and Berlin Institute of Health, Berlin, Germany;; 9 Department of Non-Communicable Disease Epidemiology, Faculty of Epidemiology and Population Health, London School of Hygiene and Tropical Medicine, London, UK;; 10 Heidelberg Institute of Global Health, Universitaetsklinikum Heidelberg, Heidelberg, Germany

**Keywords:** eating patterns, meal frequency, carbohydrate-dense snacks, nutrition transition, sub-Saharan Africa, migrants

## Abstract

**Background:**

African populations in sub-Saharan Africa and African migrants in Europe are facing a rapid upsurge in obesity. This trend has been related to urbanization, migration and associated shifts in lifestyle, including dietary habits. Whether changes in eating patterns contribute to the rising burden of obesity among African populations is currently unknown.

**Objective:**

Our aims in conducting this study were to characterize eating patterns among Ghanaian adults living in their country of origin and in Europe and to explore associations of meal patterns with body mass index (BMI).

**Design:**

Within the cross-sectional RODAM (Research on Obesity and Diabetes among African Migrants) study, data of single 24-h dietary recalls from Ghanaian adults in rural Ghana (*n* = 20), urban Ghana (*n* = 42), and Europe (*n* = 172) were recorded. Eating frequencies, energy intake, and macronutrient composition of eating occasions (EOs, i.e. meals or snacks) were compared between study sites based on descriptive statistics and *χ*^2^-/Kruskal–Wallis tests.

**Results:**

A rising gradient of EO frequencies from rural Ghana through urban Ghana to Europe was observed, mainly reflecting the differences in snacking frequencies (≥1 snack per day: 20 vs. 48 vs. 52%, *P* = 0.008). Meal frequencies were similar across study sites (≥3 meals per day: 30 vs. 33 vs. 38%, *P* = 0.80). Meals were rich in carbohydrates (median 54.5, interquartile range (IQR): 43.2–64.0 energy%) and total fats (median: 27.0, IQR: 19.9–34.4 energy %); their protein content was lowest in rural Ghana, followed by urban Ghana and Europe (*P* = 0.0005). Snacks mainly contained carbohydrates (median: 75.7, IQR: 61.0–89.2 energy%). In linear regression analyses, there was a non-significant trend for an inverse association between snacking frequencies and BMI.

**Discussion and conclusions:**

The observed integration of carbohydrate-dense snacks into the diet supports the growing evidence for a nutrition transition among African populations undergoing socioeconomic development. This analysis constitutes a starting point to further investigate the nutritional implications of increased snacking frequencies on obesity and metabolic health in these African populations.

## Popular scientific summary

- We characterized eating patterns among Ghanaian adults living in their country of origin and in Europe.
- Geographical differences in eating patterns suggest that carbohydrate-dense snacks are added to the diet upon migration and urbanization, while meal frequencies are maintained.
- Our data supports the growing evidence of a nutrition transition in African populations undergoing economic development and provides the basis to investigate the impact of shifting eating patterns on the metabolic health in these populations.

Overweight and obesity are on the rise, not just in high-income countries, but every region of the world (1). In 2015, an estimated 107.7 million children and 603.7 million adults were obese (2). Excess body weight accounted for about 4 million deaths and 120 million disability-adjusted life-years worldwide (2). The rapid increase in being overweight and obese across the African continent, in particular sub-Saharan Africa, is increasingly being recognized as a major public health concern (3–7).

Chronic-noncommunicable diseases, such as type 2 diabetes, cardiovascular diseases and certain types of cancer are associated with overweight and obesity (8–10) and are likely to further emerge in sub-Saharan Africa if no prevention strategies are implemented. It is worth noting that the highest future increase in the number of diabetes cases worldwide is expected to be in Africa, with 47.1 million adults estimated to be living with diabetes in the African Region by 2045 (11).

Overweight and obesity in sub-Saharan Africa varies widely, with a higher prevalence being reported in urban compared to rural settings (4, 5). In Ghana, for example, the prevalence of obesity among adults has reached 2–8% in rural areas and 7–34% in urban areas (2). Moreover, sub-Saharan African migrants in Europe show a higher prevalence of obesity as compared to their European host populations (12, 13). Among Ghanaian migrants, 14–54% are obese (3).

Clearly, there is a fundamental need to identify the factors driving the obesity epidemic in these populations in order to inform effective public health prevention and intervention strategies. Poor nutrition is a major modifiable risk factor for adiposity and diet-related noncommunicable diseases (10). Both urbanization in sub-Saharan Africa and migration to Europe are associated with lifestyle changes such as the adoption of unhealthy dietary habits, which may contribute to the high prevalence of obesity among sub-Saharan African populations (14, 15). The change in dietary habits from ‘traditional’ to ‘modernized’ which accompanies socioeconomic development is generally termed ‘nutrition transition’. This transition is characterized by changes in food processing, food availability, nutrient composition of foods, and overall eating patterns (14, 16). The latter is reflected by bigger meal sizes, higher eating frequencies (with an upward shift in snacking), and consuming more energy-dense foods (17–19).

The nutrition transition in sub-Saharan Africa and among African migrants in Europe has been insufficiently explored (15, 20–26). Previous research focused on the identification of dietary patterns (i.e. food groups) in indigenous and migrant Africans, and their association with metabolic health (15, 27, 28). However, much less attention has been paid to eating patterns, that is dietary habits at the level of main meals (e.g. breakfast, lunch, dinner) and snacks (smaller meals) (29). Understanding the frequency and periodicity of eating, as well as the nutritional composition of meals and snacks, could aid in gaining a deeper understanding of diet–disease relationships (30). Changes in eating patterns could potentially play a role in the observed prevalence of obesity in sub-Saharan Africa populations. While some studies suggest that eating several small portions throughout the day may benefit body weight management (31), others show the opposite (32–35). Thus, there is an ongoing controversy, and consequently there are no comprehensive science-based dietary guidelines on optimal eating and/or snacking frequencies (36). Moreover, quantitative studies dealing with eating patterns in sub-Saharan Africa and African migrants are entirely missing.

Therefore, the main aims of this study were to explore whether migration and urbanization among Ghanaian adults living in rural Ghana, urban Ghana, and Europe are associated with changes in eating patterns, and to explore the role of eating patterns for body mass index (BMI) status. The specific objectives of our study were (1) to describe the frequencies, the energy content, and the nutrient compositions of eating occasions (EO), (2) to identify differences in eating patterns between geographic locations, and (3) to explore relationships between eating patterns and BMI.

## Materials and methods

### Study design and population

The data were derived from the RODAM (Research on Obesity and Diabetes among African Migrants) study. Its conceptual framework, design, and methods are described elsewhere (37). In brief, RODAM is a multi-centre cross-sectional study carried out between 2012 and 2015 among a homogenous group of adult Ghanaians (aged 25–70 years) living in rural Ghana, urban Ghana, and Europe (London, Berlin, Amsterdam). The major aim of the RODAM Study was to segregate the relative contributions of genetic and non-genetic risk factors for obesity and type 2 diabetes in this Ghanaian study population. Ghanaian adults were defined as originating from the Ashanti region or who reported to be of Akan ethnicity. In Ghana, census data from 2010 were used to randomly select participants living in urban and rural areas of the Ashanti region. In London and Berlin, Ghanaian organizations, including churches and social unions, served as the sampling frame. In Amsterdam, Ghanaian participants were randomly drawn from the Amsterdam Municipal Register. From those invited, 76% in rural Ghana, 74% in urban Ghana, 75% in London, and 68% in Berlin participated. In Amsterdam, 67% of those invited responded and of these 53% participated in the study (3).

The study was conducted in accordance with the latest version of the Declaration of Helsinki, and the protocol was approved by the local Ethics Committees in Kumasi (CHRPE/AP/200/12), Amsterdam (W12_062 # 12.17.0086), London (6208), and Berlin (EA1/307/12). Owing to the observational study design, we did not register the RODAM study in a clinical trials registry. All participants gave informed written consent.

**Fig. 1:**
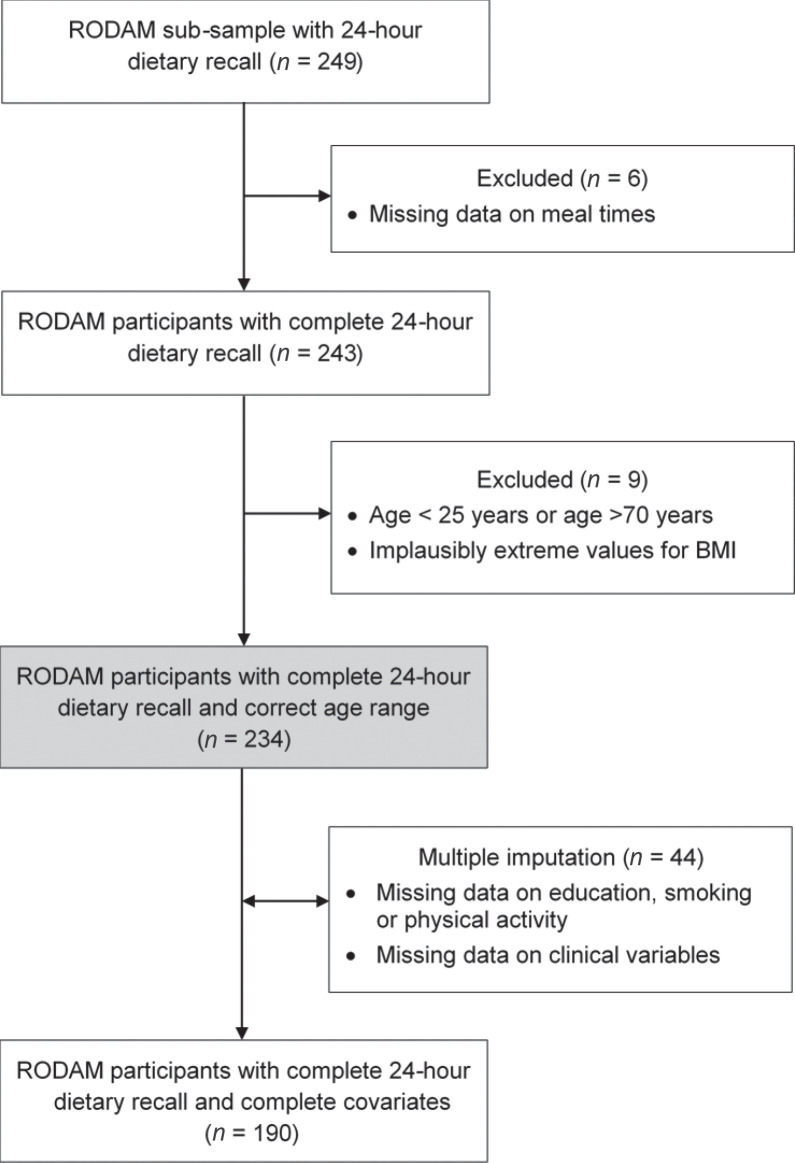
Flow chart of excluded participants and replacement of missing values by multiple imputation. RODAM, Research on Obesity and Diabetes among African Migrants.

Overall, 5,898 Ghanaians were recruited during the course of the RODAM study (2012–2015). For the present analysis, single 24 h dietary recalls (24hDRs, *n* = 249) were recorded in sub-samples of the main RODAM study population. We aimed at 100 randomly selected individuals per study site, and thus, every 12^th^ RODAM participant was invited to the recall interview. Fig. 1 summarizes the exclusion protocol and defines the analytical sample. Missing values were seen in 44 participants. Multiple imputation was applied to estimate these missing values using the fully conditional specification (FCS) method (number of imputed datasets = 5, relative efficiency >97%) (38). Data collection comprised questionnaire-based interviews and a physical examination. In addition, biological samples were drawn for biochemical analysis. Data collection was standardized and the study personnel was trained according to standard operational procedures. Interviews were conducted in the preferred language of the participant either in English, German, Dutch, or in one of the Ghanaian languages (mainly Akan).

### Assessment of eating patterns

A single 24hDR was recorded in a random sample of RODAM study participants (*n* = 249). The interview-based 24hDR was carried out by trained staff in face-to-face interviews, according to the 5-Step Multiple Pass Method (39). Participants were asked to provide information about eating times, types of foods and beverages consumed, portion sizes, and specific information about brands and recipes. One single 24hDR was recorded on a weekday. For this, a suitcase with common Ghanaian household utensils was provided to the interviewers to facilitate the standardized estimation of portion sizes with familiar and uniform cooking equipment. When participants did not provide portion sizes, site-specific pre-defined portion sizes were assigned. The German Nutrient Database (BLS 3.01) (2010) and the West African Food Composition Table (2012) were used to translate food intake (g/day) into energy consumption (kcal/day) and intake of macronutrients (% energy from carbohydrates, protein and fat), fibre (g/day), and alcohol (g/day). In addition, a Ghana-Food Propensity Questionnaire (Ghana-FPQ) was administered to all participants, capturing the usual intake frequencies of 134 pre-defined food items during the previous 12 months (15). The Ghana-FPQ was used to rank the participants according to their usual energy intake, which cannot be produced from one 24hDR due to intra-individual variation of the diet.

For the calculation of eating frequencies, we used 24hDR data and compared the feasibility of different definitions for EOs, that is meals and snacks. In the final analysis, we applied a modified version of the definition proposed by Leech et al. (29) and Murakami and Livingstone (40), as this approach best fits our data. It is widely practiced and therefore, facilitates comparability with other studies (41–43). An individual EO was defined as any occasion where food and/or beverages were consumed, with a time interval of at least 30 min to separate preceding and succeeding EOs. An EO had a minimum energy intake of 210 kJ. The classification of meals and snacks according to traditional meals (e.g. breakfast, lunch, dinner) and snacking times did not reflect eating patterns in the RODAM study population (Supplementary Information, Fig. S2). Therefore, EOs were divided into meals and snacks according to their contribution to daily energy intake. Snacks were defined as every EO contributing <15% to the total daily energy intake, whereas all other EOs were classified as meals.

### Assessment of socio-demographic and lifestyle factors

Information regarding demographics (age, sex, study site), socio-economic status (education), health status (self-reported history of diabetes), and lifestyle factors (smoking, physical activity) was obtained from a general questionnaire that was either self-administered or applied in face-to-face interviews. The educational status was categorized as follows: never been to school or elementary school; lower vocational schooling or lower secondary schooling; intermediate vocational schooling or intermediate/higher secondary schooling; and higher vocational schooling or university. Smoking was assessed based on the question ‘Do you smoke at all?’; the smoking status was categorized as current and former smokers or non-smokers. The WHO Global Physical Activity Questionnaire (44) was used to derive physical activity (hours/week), including the three domains: physical activity at work, travel to and from places, as well as recreational activities.

### Anthropometric measurements

Participants were invited to take part in a physical examination, including anthropometric measurements. Precisely, height and weight were measured in light clothing and without shoes, using a portable stadiometer (SECA 217) and a digital scale (SECA 877, SECA, Hamburg, Germany), respectively. BMI was calculated as weight/(height)^2^ (kg/m^2^). These measurements were performed in duplicates and means were taken for analysis.

### Statistical analyses

For the present study population, general characteristics and the nutrient composition of EOs, meals, and snacks are presented as means (standard deviations, SD) for normally distributed variables, and as medians (interquartile ranges) for non-normally distributed variables. For categorical data, we present percentages. Moreover, categories of EOs, meals, and snacking frequencies were constructed (1–2; 3; ≥ 4 EOs per day; 1; 2; ≥3 meals per day; 0; 1; ≥2 snacks per day) and the distributions of general characteristics across these categories were calculated. The frequencies of EOs and continuous characteristics were compared across study sites by making use of the *χ* ^2^-test and the Kruskal-Wallis test, respectively. We estimated misreporting as the ratio of energy intake (kcal/d) and Estimated Energy Requirements (EER, kcal/d). EER was calculated as the product of Basal Metabolic Rate (BMR) and physical activity level (PAL), as follows:

EER (men) = [10× weight [kg] + 6.25× height [cm] – 5× age [years] + 5] × PAL

EER (women) = [10× weight [kg] + 6.25× height [cm] – 5× age [years] – 161] × PAL.

We defined the boundaries for misreporting as mean energy intake-to-EER ratio ±1.5× SD of the energy intake-to-EER ratio. Mean bias in our study population was calculated as [(mean energy intake – mean EER)/mean EER].

Associations between frequencies for EOs, meals, and snacks were investigated by making use of linear regression analyses (Supplementary Information, Fig. S4). For the associations of meal frequencies and snacking frequencies with BMI, multiple-adjusted linear regression analyses were performed. A combined analysis for rural Ghana, urban Ghana, and Europe was conducted due to the small study population (*n* = 234). Both exposures were entered as categorical variables, using the most common frequency as the reference: 2 meals per day and 0 snacks per day, respectively. The PROC REG procedure in SAS was used to calculate the adjusted regression coefficients with their 95% confidence intervals for BMI by one increase of meal and snacking frequency. Potential confounding factors were considered as follows: Model 1 was adjusted for important demographic risk factors of obesity (age, sex, study site), and Model 2 additionally included established confounders of the association between diet and obesity, namely socio-economic and lifestyle factors (education, smoking, physical activity). To account for suggested effects beyond increased energy intake (34), Model 3 accounted for energy intake (kcal/d).

All statistical analyses were performed by using SAS (version 9.3; SAS Institute Inc., Cary NC), except for the linear regression analysis investigating associations between frequencies of EOs, meals, and snacks. For the latter, Graph Pad Prism (version 8.2.0) was used. Figures were created either in Graph Pad Prism or OriginPro2016 (Origin Lab Corporation, Northampton, USA).

## Results

### Study population

General characteristics of the RODAM study participants with complete 24hDRs included in the present analysis (*n* = 234) are shown in Table 1. The characteristics for Amsterdam, London, and Berlin were similar, except for smoking status and physical activity level (median in analytical sample: 76; IQR: 21–162 vs. median in total study population: 56; IQR: 10–144). Henceforth, we combined the European sites in further analyses.

**Table 1:** General characteristics of the Research on Obesity and Diabetes among African Migrants (RODAM) study participants with complete 24 h dietary recalls (*n* = 234) included in the present analysis

	All (*n* = 234)	Rural Ghana (*n* = 20)	Urban Ghana (*n* = 42)	Europe (*n* = 142)		
				Amsterdam (*n* = 97)	London (*n* = 21)	Berlin (*n* = 54)
Sex (male)	42.7	50.0	33.3	42.3	47.6	46.3
Age (years)	45.2 ± 10.3	41.4 ± 14.7	46.1 ± 11.9	47.2 ± 8.3	46.0 ± 9.7	42.3 ± 9.6
Education						
None	26.1	45.0	35.7	27.8	9.5	14.8
Lower	39.3	25.0	47.6	42.3	28.6	37.0
Intermediate	20.9	15.0	11.9	22.7	23.8	25.9
Higher	13.7	15.0	4.8	7.2	38.1	22.2
Former or current smoker (%)	8.6	5.0	4.8	9.3	0.0	14.8
Energy intake (kcal/d)	1,801 (1,242–2,268)	2,030 (1,262–2,461)	1,905 (1,566–2,512)	1,672 (1,098–2,258)	1,445 (890–2,098)	1801 (1,311–2,193)
Carbohydrate (energy %)	59.4 (50.2–67.4)	58.0 (49.7–69.0)	66.1 (58.9–72.0)	58.2 (49.6–65.4)	62.2 (52.8–70.1)	55.9 (48.4–62.3)
Protein (energy %)	16.2 (12.3–18.9)	13.5 (11.7–15.6)	14.0 (11.3–17.7)	16.6 (12.8–19.6)	14.7 (11.5–16.7)	17.8 (14.1–21.1)
Fat (energy %)	23.0 (17.9–30.6)	29.0 (20.7–35.0)	20.1 (15.9–25.4)	22.9 (17.6–24.4)	21.9 (18.9–33.7)	26.4 (20.1–31.2)
Fibre (g/d)	20.7 (13.7–29.1)	20.8 (14.6–32.0)	25.0 (15.9–30.3)	20.2 (13.0–30.6)	20.7 (11.6–29.3)	19.6 (14.3–24.3)
Physical activity (h/week)	20.5 (6.0–39.0)	15.4 (9.8–27.3)	20.5 (7.0–38.0)	29.1 (7.4–42.5)	4.3 (2.0–25.7)	15.1 (4.7–40.0)
Body mass index (BMI) (kg/m^2^)	27.4 ± 4.7	23.5 ± 3.8	28.1 ± 5.1	27.7 ± 4.5	29.0 ± 4.1	27.0 ± 4.5

Data for continuous, normally distributed variables are shown as means ± standard deviations, for non-normally distributed variables as medians (percentile 25 – percentile 75), and for categorical variables as percentages.

Briefly, the majority of participants were female (57.3%) and middle-aged (mean: 45.2 ± 10.3 years). Participants in Europe were mainly first-generation migrants (99.2%). They had the highest degree of education, and were more frequently former or current smokers than their counterparts in Ghana. There were three night-workers in our study population, two in London, and one in Amsterdam. The mean total daily energy intake was lowest in Europe and at a similar level between rural Ghana and urban Ghana. BMI and physical activity were lowest in rural Ghana (mean BMI: 23.5 kg/m^2^) and at a similar level between urban Ghana and Europe (mean BMI, urban Ghana: 28.1 kg/m^2^; mean BMI, Europe: 27.7 kg/m^2^).

For a comparison of general characteristics between the RODAM sub-sample used for the present analysis and the total RODAM study population the reader can refer to the Supplemental Material (Table S1). Briefly, general characteristics were similar between the analytical sample and the total study population, except for discrepancies in physical activity levels.

### Frequencies of EOs, meals and snacks across study sites

**Fig. 2:**
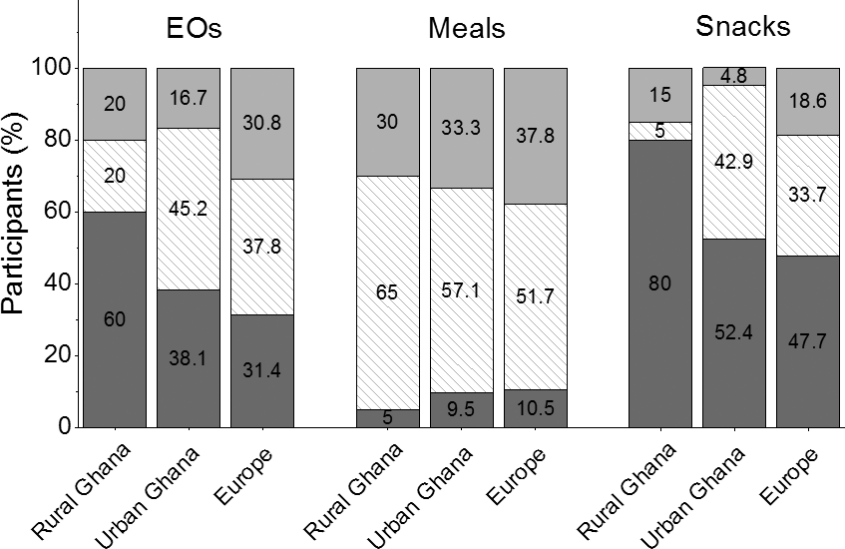
Distribution of eating frequencies, meals and snacks across study sites. The bars indicate the percentages of participants in the frequency categories for eating occasions (EOs), meals, and snacks according to study site. Lowest frequency categories are shown in dark grey, intermediate frequency categories are shown in dashed, and highest frequency categories are shown in light grey. EO categories: 1–2, 3, ≥4 per day; meal categories: 1, 2, ≥3 per day; snack categories: 0, 1, ≥2 per day.

Figure 2 shows the proportions of participants in frequency categories of EOs (1–2, 3, ≥4), meals (1, 2, ≥3), and snacks (0, 1, ≥2) across study sites. For EO frequencies, a rising gradient from rural Ghana through urban Ghana to migrants in Europe was seen (*P* = 0.049). In rural Ghana, 60% of the participants had 1–2 EOs per day, while in urban Ghana, the majority of participants (62%) reported ≥3 EOs per day. In Europe, one-third each consumed 1–2, 3, or ≥4 EOs per day.

Meal frequencies in rural Ghana, urban Ghana, and Europe were similar (*P* = 0.80), with the highest proportion of participants consuming two meals per day. The proportion of participants consuming more than two meals per day was lowest in rural Ghana (30%) and highest in Europe (38%) (difference not significant).

Snacking frequencies differed across study sites, reflecting the same trend as for EO frequencies. The highest proportion of individuals consuming snacks was seen in Europe, followed by urban Ghana and rural Ghana (*P* = 0.008). A total of 80% of the participants in rural Ghana and around half of the participants in urban Ghana and Europe did not snack at all.

Linear regression analysis showed a strong correlation between EO and snacking frequencies (Pearson’s correlation *r* ^2^ = 0.57). In contrast, there was no correlation between EO and meal frequencies (Pearson’s correlation *r* ^2^ = 0.02, Supplementary Material, Fig. S4).

### Energy contribution and nutrient compositions of meals and snacks across study sites

Nutrient compositions of meals and snacks for the total study population and across study sites are shown in Table 2. Overall, snacks provided a higher proportion of energy from carbohydrates as compared to meals (76% vs. 55%) and, correspondingly, a lower proportion of energy from protein and fat. Also, snacks were characterized by a generally lower fibre intake as compared to meals.

**Table 2:** Energy content and nutrient composition of meals and snacks across study sites

	Total	Rural Ghana	Urban Ghana	Europe	*P*
**Meals**					
n participants	234	20	42	172	
Energy (kcal/d)	714 (500–986)	863 (631–1,084)	844 (548–1,176)	668 (474–928)	0.006
Carbohydrates (energy %)	54.5 (43.2–64.0)	58.1 (44.8–69.0)	61.8 (50.4–69.8)	52.3 (41.3–62.1)	0.003
Protein (energy %)	17.2 (12.9–21.7)	14.1 (11.7–16.0)	14.7 (11.3–18.2)	18.1 (13.9–23.2)	0.0005
Fat (energy %)	27.0 (19.9–34.4)	28.8 (17.3–37.2)	21.4 (17.9–31.3)	27.5 (20.9–34.6)	0.052
Fiber (g/d)	8.1 (6.2–11.3)	9.0 (6.9–11.3)	9.2 (7.2–12.7)	7.8 (5.6–11.0)	0.039
**Snacks**					
n participants	114	4	20	90	
Energy (kcal/d)	152 (109–240)	189 (151–262)	211 (124–280)	140 (96–222)	0.053
Carbohydrates (energy %)	75.7 (61.0–89.2)	63.9 (49.7–76.6)	80.1 (70.6–88.9)	74.9 (57.8–89.2)	0.207
Protein (energy %)	9.2 (5.3–13.4)	11.3 (10.8–12.3)	9.2 (6.9–13.8)	9.1 (5.3–13.4)	0.539
Fat (energy %)	12.1 (3.8–24.5)	24.3 (12.3–38.3)	8.2 (3.3–15.5)	12.2 (3.7–24.7)	0.165
Fiber (g/d)	2.6 (1.4–4.2)	4.1 (2.2–6.2)	2.6 (1.3–5.4)	2.6 (1.4–4.1)	0.516

Data are presented as medians (percentile 25-percentile 75). *P*-values for differences across study sites were calculated by making use of Kruskal–Wallis tests.

Across study sites, meals differed in their nutrient content: energy intake from protein was higher in Europe (median: 18.1 energy%) than in urban Ghana (median: 14.7 energy%) or rural Ghana (median: 14.1 energy%). In urban Ghana, energy intake from total fat was lowest (median: 21.4 energy%) but energy intake from carbohydrates was highest (median: 61.8 energy%). Europe showed the lowest fibre intake for meals.

With respect to snacks, their nutrient content was similar across study sites. The contribution of snacks to daily energy intake was highest in Europe (8.2%), followed by urban Ghana (6.1%) and rural Ghana (3.2%).

### Characteristics of participants across categories of EOs, meals and snacks

General characteristics across frequency categories of EOs, meals, and snacks are presented in Table 3. The mean age was similar across all frequency categories. Trends across categories of snacking and EO frequencies were comparable and the following was noted: Participants with higher EO and snacking frequencies were more often men and non-smokers. They showed higher educational attainment, higher daily energy intakes, higher physical activity, and in the case of EOs, lower BMI than participants with lower EO and snacking frequencies.

**Table 3:** General characteristics across frequencies of eating occasions, meals, and snacks

	Number of eating occasions			Number of meals			Number of snacks		
	1–2	3	≥4	1	2	≥3	0	1	≥2
*n*	82	88	64	23	126	85	120	77	37
Male sex	31.7	45.5	53.1	47.8	39.7	45.9	38.3	39.0	64.9
Age (years)	44.3 ± 11.0	46.0 ± 9.8	45.4 ± 10.0	44.5 ± 10.5	45.4± 10.1	45.1± 10.6	44.7± 10.9	45.9 ± 9.7	45.5 ± 9.4
Education									
None	29.0	22.7	22.5	34.8	23.8	23.8	24.8	27.3	20.0
Lower	44.9	40.9	35.9	26.1	44.3	40.0	46.5	35.1	35.1
Intermediate	19.5	19.6	24.4	30.4	18.7	21.4	19.2	19.7	28.7
Higher	6.6	16.8	17.2	8.7	13.2	14.8	9.5	17.9	16.2
Smoking (current/quit)	9.8	8.4	7.8	13.0	6.8	10.3	10.7	7.3	5.4
Energy intake (kcal/d)	1,427 ± 652	1,987 ± 727	2,178 ± 780	1,727 ± 819	1,705 ± 717	1,987 ± 823	1,584 ± 725	1,980 ± 808	2,188 ± 628
Misreporting (Energy intake/Estimated energy requirement [EI/EER])	0.87 (0.54–1.19)	1.07 (0.83–1.41)	1.13 (0.92–1.46)	0.98 (0.57–1.45)	1.03 (0.75–1.30)	1.08 (0.86–1.35)	0.93 (0.60–1.20)	1.09 (0.84–1.42)	1.22 (0.92–1.59)
Physical activity (h/week)	14.2 (3.2–30.0)	19.9 (5.6–41.2)	28.3 (9.5–43.7)	12.9 (3.3–43.7)	20.0 (6.3–38.2)	19.8 (6.5–39.2)	15.2 (5.6–31.0)	20.8 (3.7–40.6)	35.6 (17.6–47.3)
Body mass index (BMI) (kg/m^2^)	28.0 ± 5.1	27.4 ± 4.9	26.6 ± 3.7	27.1 ± 4.6	27.6 ± 4.7	27.1 ± 4.7	27.1 ± 5.2	27.6 ± 4.3	26.1 ± 3.7

Data for continuous, normally distributed variables are shown as means ± standard deviations, for non-normally distributed variables as medians (percentile 25-percentile 75), and for categorical variables as percentages.

EI, Energy Intake; EER, Estimated Energy Requirement.

For meal frequencies, participants in the highest and the lowest category of meal frequency were more often men and former or current smokers as compared to those in the medium category. Participants in the highest category of meal frequency were more frequently better educated, had higher daily energy intake, and the highest level of physical activity compared to their counterparts in the lower categories. Mean BMI was similar across categories of meal frequency.

Misreporting defined as energy intake over EER was higher in higher tertiles of EOs, meals, and snacks, respectively. The boundaries for misreporting ranged from 0.33 to 1.85, translating into 11 under-reporters, 21 over-reporters, and 202 plausible reporters. The mean bias was 0.06.

### Associations of eating patterns with BMI

Table 4 shows the associations of meals and snacking frequencies with BMI. In the model adjusted for age, sex, and study site (Model 1), snacking tended to be associated with lower BMI, but this was not significant. Further adjustment for education, smoking, and physical activity (Model 2), as well as energy intake (Model 3, full model), slightly attenuated the observed point estimates. Regarding meal frequencies, an A-shaped association with BMI was observed in the basic Model 1, and after further adjustment for confounders (Model 2) and/or mediators (Model 3). None of the models was statistically significant.

**Table 4:** Associations of meals frequencies and snack frequencies with body mass index (kg/m^2^)

Model	kg/m^2^ change (95% confidence intervals)					
	1 meal per day	2 meals per day	≥3 meals per day	0 snacks per day	1 snack per day	≥2 snacks per day
*n*	23	126	85	120	77	37
Model 1	−0.36 (−2.27, 1.54)	Reference	−0.40 (−1.57, 0.78)	Reference	−0.72 (−1.96, 0.52)	−1.02 (−2.65, 0.60)
Model 2	−0.45 (−2.37, 1.46)	Reference	−0.44 (−1.62, 0.74)	Reference	−0.71 (−1.96, 0.55)	−0.99 (−2.67, 0.69)
Model 3	−0.44 (−2.35, 1.48)	Reference	−0.33 (−1.53, 0.87)	Reference	−0.58 (−1.88, 0.72)	−0.82 (−2.57, 0.92)

β-coefficients (kg/m^2^ change) and 95% confidence intervals (CIs) were calculated by linear regression analyses.

Model 1: adjusted for age (years), sex (male/female), study site (rural Ghana, urban Ghana, Amsterdam, London, Europe).

Model 2: Model 1 + education (4 categories), smoking (3 categories), physical activity (hours/week).

Model 3: Model 2 + energy intake (kcal/d).

## Discussion

### Summary of main results

In summary, eating frequencies and macronutrient intakes differed between rural and urban Ghana and between Ghana and Europe. Our data suggests that carbohydrate-dense snacks are added to the diet, following urbanization in sub-Saharan Africa and migration to Europe. Across study sites, snacks were similar in their nutrient composition, whereas meals differed in their protein content. Energy intake from protein in meals was highest in Europe, followed by urban Ghana and rural Ghana. Higher snacking frequencies tended to be associated with lower BMI, but this was not statistically significant.

### Eating patterns and nutrition transition

Differences in eating patterns between Ghanaians living in rural Ghana, urban Ghana, and Europe are in accordance with previous descriptions of an ongoing nutrition transition among these populations (15, 16). More specifically, this work and previous studies in African migrants in Europe indicate that certain aspects of the original dietary habits are preserved, while some dietary habits of the host population were adopted (21, 25, 26, 45, 46). Dietary pattern analysis for the RODAM study population showed that core food groups and combinations thereof were maintained even several years after migration and upon acculturation. Core food groups of the diet in Ghana included starchy roots, tubers and plantain with palm oil- as well as peanut-based soups and stews. Moreover, fish and goat meat were frequently consumed across study sites, while fruits and dairy intakes were low (15, 28).

Previous studies about eating patterns in African migrants have been qualitative in nature (21, 23, 26), but still support the findings from the present study. Osei-Kwasi et al. studied the influence of migration on dietary practices of Ghanaians living in the UK using face-to-face interviews (*n* = 31) (23). Participants retained, to a varying degree, some aspects of Ghanaian dietary practices, whilst adopting key features of the UK food culture. The majority of participants showed only limited adoption of UK dietary patterns and consumed 1–2 meals per day, which agrees with our findings. Tuomainen (26) investigated how eating patterns of Ghanaian migrants evolved among 18 Ghanaian households in London in a qualitative study. They found that Ghanaian migrants in London consumed fewer meals per day compared to what they consumed in Ghana (26). This contrasts the stable meal frequencies across the locations in the present analysis. The consumption of mainly three meals per day in urban Ghana has also been reported by Frank et al. (47) and Nti (48), whereas Plahar et al. reported the consumption of two meals per day in Southern Ghana (49). The reason for this inconsistency requires further investigation. However, comparisons should generally be made with caution, since the term ‘meal’ was not further defined and methods of dietary assessment were diverse in these earlier studies.

Regarding the consumption of snacks, in our study, only few participants in rural Ghana consumed snacks, while snacking was more common in urban Ghana and Europe. These results are in line with Tuomainen’s study reporting that young British Ghanaian adults have ‘acquired a taste for snacks’ (50). Also, Gibson et al. have reported increased intakes of carbohydrate-dense confectionery among Ghanaian adults living in Accra as compared to those living in London (22). Indeed, in urban Ghana, typical snacks comprise nuts and seeds, and savoury biscuits with meat. Among Ghanaian migrants in Europe, cakes and sweets, bread with sweet spread or condiments, and sodas and juices dominate (24). Moreover, snacking was uncommon among first-generation migrants from Ghana (23), illustrating the role of the length of stay for changes in eating patterns. A higher consumption of snacks after migration has also been reported in other migrant populations (51–54), which is in support of our results. In fact, Koctuerk found that snacking increases upon migration and acculturation (55). In the RODAM study population, European residency was related to higher education, which may be associated with a busier lifestyle and the adoption of snacking behaviour. Other factors can likewise play a role in the process of dietary acculturation (56): The importance of cultural identity, taste preferences, health beliefs, availability and cost of traditional foods, and having Ghanaian social networks have been highlighted (21, 57–59).

### Eating patterns and BMI

There has been an ongoing debate about optimal eating frequencies throughout the scientific literature. The majority of published cross-sectional studies have reported inverse relationships between eating frequency and measures of obesity (reviewed in (31, 60–62)). This tendency – even though insignificant – was also seen in our study. However, it should be noted that an inverse relationship between eating frequencies and BMI/obesity has not been found in all studies (63–65); and published weight-loss interventions have mainly reported null findings (33).

Several mechanisms have been proposed by which increased eating frequencies may impact body weight (34, 66). Flatter hunger profiles, more even glucose and insulin levels, and prolonged gastric emptying have been associated with increased snacking frequencies, thus helping to adjust both portion sizes and eating frequency of subsequent meals (66–69). On the other hand, snacking might not be satiating, creating more opportunities to eat and may lead to increased energy intake (34).

RODAM participants in higher categories of snacking and eating frequency reported a higher energy intake despite lower BMIs as compared to participants in lower frequency categories. Our results are in line with a recent meta-analysis of 10 cross-sectional studies showing that increased eating frequency is associated with lower obesity risk, but higher energy intake (31).

These observations may at first be counterintuitive, however, higher eating frequencies have been suggested to be more compatible with an active lifestyle (53), which is supported by the present study. Indeed, the frequently observed inverse relationships between eating frequencies and adiposity in cross-sectional studies have been suggested to be an artefact due to a disregard of physical activity as an important confounder (70). Model adjustment for physical activity in this study slightly attenuated the estimates. Still, further research in larger populations is needed for a better understanding of the relationships between eating frequency and obesity. In particular, additional information about the quality of macronutrients from improved nutrient databases of African foods is needed to evaluate the impact of snacking on the overall nutrient content of the diet.

### Strengths and limitations

The vast majority of the participants in our study came from the Ashanti Region in Ghana. This homogeneity in terms of geographic origin constitutes a major strength of our work in terms of representativeness and generalizability. Moreover, standardized procedures for data collection across study sites have been applied, limiting the measurement error and improving the precision of the data. The assessment of dietary intake was culture-specific and detailed. Nutrient compositions of meals and snacks were calculated according to European and West African-specific food composition databases, which provided high-quality dietary data. However, inter-interviewer and intra-participant variability may have occurred, limiting the ability to capture the day-to-day variability in dietary behaviour in terms of absolute intake amounts and usual eating frequencies for each participant. Also, underreporting by both instruments, the Ghana-FPQ and the single 24hDR, cannot be fully excluded. These limitations might have affected the estimated associations between eating frequencies and BMI in both directions. Misreporting, however, appeared to be minor in this study population. Cultural differences for the definition of meals and snacks were taken into account to avoid limitations of predefined eating concepts. Hence, we used a neutral definition for EOs, meals, and snacks, which has widely been used and allows for comparability of our findings. Fine-graded time intervals (<30 min) were not available and smaller EOs may not have been captured. The study population was small, particularly for rural Ghana, which could impair the external validity of the observed eating frequencies. However, discrepancies between the analytical sample and the full study population were minor, arguing against selection bias and for internal validity. Clearly, the observed associations between eating frequencies and BMI are exploratory and hypothesis building. We cannot infer causal relationships between eating patterns and BMI due to the cross-sectional nature of our study. Decreased eating frequencies could be either a cause or a consequence of obesity, if obese individuals skipped meals and snacks to control their body weight. To gain a better understanding of the role of meal patterns for obesity and metabolic health, prospective studies in larger populations are required.

## Conclusion

In conclusion, geographical differences in eating patterns, both across study sites in Ghana and between Ghana and European sites, contribute to the evidence for an ongoing nutrition transition in sub-Saharan Africa and upon migration to Europe. Our results suggest that urbanization and migration go along with increased consumption of carbohydrate-dense snacks, whereas meal frequencies were maintained. This work is the first quantitative study dealing with meal patterns among sub-Saharan African populations in their host country and in Europe. It constitutes a starting point to further investigate the nutritional implications of the upward shift in snacking on obesity and metabolic health among African populations undergoing transition. Dietary guidelines embracing meal patterns hold much promise to assist populations in the preparation of healthy diets. However, further research is needed to inform public health prevention and invention programs in order to tackle the obesity epidemic.

## Supplementary Material

Carbohydrate-dense snacks are a key feature of the nutrition transition among Ghanaian adults – findings from the RODAM study
