# Supplementary material for: Carbohydrate-dense snacks are a key feature of the nutrition transition among Ghanaian adults – findings from the RODAM study
Source: Food Nutr Res. 2021 May 6;65:10.29219/fnr.v65.5435. doi: 10.29219/fnr.v65.5435 (PMC8388941; doi:10.29219/fnr.v65.5435)
Supplement: Carbohydrate-dense snacks are a key feature of the nutrition transition among Ghanaian adults – findings from the RODAM study [file FNR-65-5435-s001.docx]

**List of abbreviations:**

BLS Bundeslebensmittelschlüssel (German Nutrient Database)

BMI Body Mass Index

EO Eating occasion

FCS Fully conditional specification

Ghana-FPQ Ghana-Food Propensity Questionnaire

IQR Interquartile range

RODAM Research on Obesity and Diabetes among Africa Migrants

UK United Kingdom

WHO World Health Organization

24hDR 24-hour dietary recall
